# Serotype Distribution and Drug Resistance in *Streptococcus pneumoniae*, Palestinian Territories

**DOI:** 10.3201/eid1701.100886

**Published:** 2011-01

**Authors:** Randa Kattan, Amal Abu Rayyan, Inas Zheiman, Suzan Idkeidek, Sabri Baraghithi, Nabeel Rishmawi, Sultan Turkuman, Afaf Abu-Diab, Riyad Ghneim, Madeleine Zoughbi, Rula Dauodi, Raed Ghneim, Abed-El-Razeq Issa, Issa Siryani, Randa Al Qas, Rawan Liddawi, Hatem Khamash, Moein Kanaan, Hiyam Marzouqa, Musa Y. Hindiyeh

**Affiliations:** Author affiliations: Caritas Baby Hospital, Bethlehem, Palestinian Territories, West Bank (R. Kattan, N. Rishmawi, S. Turkuman, A. Abu-Diab, Riyad Ghneim, M. Zoughbi, R. Dauodi, Raed Ghneim, A.-E.-R. Issa, I. Siriani, R. Al Qas, R. Lidawi, H. Marzouqa, M.Y. Hindiyeh);; Bethlehem University, Bethlehem (A. Abu Rayan, M. Kanaan, M.Y. Hindiyeh);; Maqassed Islamic Hospital, East Jerusalem, Palestinian Territories (I. Zheiman, S. Idkeidk, S. Barghithi, H. Khamash)

**Keywords:** Streptococcus pneumoniae, antimicrobial drug resistance, vaccine, PCV7, PCV13, bacteria, children, West Bank, Palestinian Territories, dispatch

## Abstract

To determine antimicrobial drug resistance of *Streptococcus pneumoniae* serotypes, we analyzed isolates from blood cultures of sick children residing in the West Bank before initiation of pneumococcal vaccination. Of 120 serotypes isolated, 50.8%, 73.3%, and 80.8% of the bacteremia cases could have been prevented by pneumococcal conjugate vaccines. Serotype 14 was the most drug-resistant serotype isolated.

*Streptococcus pneumoniae* infection is a common cause of illness and death worldwide, responsible for an estimated 1.2 million infant deaths each year (*1*). The polysaccharide capsule is one of the primary virulence factors that contributes to *S. pneumoniae* pathogenicity (*2*). Management of *S. pneumoniae* infections has been complicated by the emergence of multiple antimicrobial drug–resistant strains (*3*).

Before the introduction of the 7-valent polysaccharide-protein conjugate vaccine (PCV7), serotypes included in this vaccine were responsible for ≈90% of pneumococcal infections in children living in industrialized countries; in developing countries, coverage has been reported as low as 26% (*4**,**5*). The use of PCV7 reduced the incidence of invasive pneumococcal disease (IPD) in children <5 years of age in the United States by 76% (*6*). However, nonvaccine serotypes (i.e., 19A, 6C) have emerged as primary pathogens in IPD (*7*). More recently, the American Advisory Committee on Immunization Practices recommended using the new Food and Drug Administration–licensed 13-valent pneumococcal conjugate vaccine (PCV13), which contains the 7 serotypes present in PCV7 and 6 additional serotypes (1, 3, 5, 6A, 7F, and 19A).

## The Study

In the Palestinian Territories, West Bank, the epidemiology of IPD is not well defined. Our study characterized the serotypes and antimicrobial drug resistance patterns of 120 consecutive *S. pneumoniae* isolates collected from blood cultures of patients admitted to Caritas Baby Hospital (n = 113) during January 2001–April 2010 or Maqassed Islamic Hospital (n = 7) during January 2009–April 2010. Both hospitals are well-equipped and are the major hospitals that perform blood cultures in the West Bank. Blood cultures are overused at both institutions; thus, isolated *S. pneumoniae* are representative of circulating serotypes. Because *S. pneumoniae* meningitis and invasive pneumonia are rare, these IPDs were not included in the study.

Blood cultures were collected from patients suspected of having sepsis or endocarditis. Criteria for collecting blood cultures included leukocytosis with left shift, elevated C-reactive protein level, fever, signs of toxicity, hypotension, and hemodynamic instability. One isolate per patient was included in the study. Patients’ ages ranged from 1 day to 11 years; most (71.7%) were <2 years of age.

All BACTEC (Becton Dickinson, Sparks, MD, USA)–positive blood culture samples were placed on 5% sheep blood agar, chocolate agar, and MacConkey agar obtained from Hy-Laboratories Ltd. (Rehovot, Israel). Suspected *S. pneumoniae* colonies were identified on the basis of colony morphologic appearance, α-hemolysis on 5% sheep blood agar, Gram stain appearance, bile solubility, and optochin susceptibility. All strains were stored at −80°C until further testing.

*S. pneumoniae* isolates were serotyped by performing a series of PCRs using primers described by Pai et al (*8*). Because the serotype 6B primer cross-reacts with the common 6A *cps* operon sequence, we did not differentiate serotypes 6A/B.

Of the 120 *S. pneumoniae* isolates, 117 (97.5%) were serotyped; 3 (2.5%) could not be typed. These results are similar to those of Pai et al., who successfully typed 95.5% of the *S. pneumoniae* stains by using the same PCR technique (*8*). In the West Bank, 20 *S. pneumoniae* serotypes were identified; serotypes 6A/B, 14, 1, and 9V were the predominant strains (Table A1). Overall, 97 (80.8%) of the serotypes are included in PCV13, and >61 (50.8%) are covered by PCV7 (6A/B not differentiated).

After serotypes were stratified by age groups, 86 (71.7%) *S*. *pneumoniae* strains were isolated from children <2 years of age; 34 (28.3%) were isolated from children >2 years of age. High *S. pneumoniae* serotype coverage by PCV13 was noted for each age group, 77.9% and 76.5%, respectively. Conversely, serotype coverage provided by PCV7 for each age group was 60.5% and 26.5%, respectively. Of the 6 additional serotypes included in PCV13 and not in PCV7, serotypes 1, 3, and 5 appeared to be common in our study population. Notably, *S. pneumoniae* serotype 1 was not seen in children <2 years of age.

Distribution of the *S. pneumoniae* serotypes was similar to that in a study by Fraser et al. in Israeli and in Bedouin children residing in southern Israel (*9*). However, unlike the study from southern Israel in which serotypes 1 and 5 predominated, in this study, serotypes 6A/B and 14 predominated. Different *S. pneumoniae* serotype distribution was also noted when serotypes isolated from the West Bank were compared with those from other regional countries such as Kuwait and Saudi Arabia (*10*).

Drug susceptibility testing of the different *S. pneumoniae* serotypes was performed by using disk diffusion on Mueller-Hinton agar supplemented with 5% sheep blood agar for penicillin (oxacillin) (1 µg), erythromycin (15 µg), ofloxacin (5 µg), co-trimoxazole (25 µg), and vancomycin (30 µg) according to the Clinical and Laboratory Standards Institute (CLSI) guidelines (*11*). In addition, penicillin and cefotaxime minimal MICs were determined for all isolates by Etest according to the manufacturer’s recommendations (AB Biodisk, Solna, Sweden). Interpretation of penicillin and cefotaxime MIC results was performed by using the CLSI nonmeningitis guidelines, (parenteral penicillin susceptible <2 µg/mL, intermediate 4 µg/mL, resistant >8 µg/mL, and for cefotaxime susceptible <1 µg/mL, intermediate 2 µg/mL, resistant >4 µg/mL), and the meningitis guidelines were applied for penicillin (parenteral penicillin susceptible <0.06 µg/mL, resistant >0.12 µg/mL) (*11*).

Overall, of the 120 *S. pneumoniae* blood isolates evaluated, 50 (41.7%) were penicillin susceptible; 70 (58.3%) were resistant based on the 1-μg oxacillin disk-diffusion results. However, MICs for penicillin showed that 118 (98.3%) isolates were susceptible and only 2 (1.7%) *S. pneumoniae* isolates were intermediately resistant (Table A1). These results are similar to those from Germany and the United States, which reported low penicillin intermediate rates of 0.2% and 5.6%, respectively (*12**,**13*). Unlike the absence of resistant isolates in this study, both countries reported 1.2% resistance rates.

The small sample size is a limitation in this study and mandates caution when comparing the results with those of other studies. The use of broth microdilution to determine penicillin MIC, as reported in the Germany study or Etest as performed in this study, has been documented to produce comparable results (*14*). The penicillin resistance rate of the *S. pneumoniae* isolates was higher after applying the CLSI meningitis guidelines (45.8% sensitive and 54.2% resistant).

Unlike the low penicillin resistance rate, higher resistance rates were noted for erythromycin and co-trimoxazole. Of the 120 *S. pneumoniae* isolates evaluated, 32 (26.7%) were resistant to erythromycin, and 53 (44.2%) were resistant to co-trimoxazole (Table A1). After stratifying erythromycin and co-trimoxazole resistance rates by serotype, we showed that serotype 14 had the highest resistance rates for both antimicrobial drugs (75%). Resistance to >1 agent was noted for 53.3% of the serotype 14 isolates (Table A1). *S. pneumoniae* erythromycin resistance in the West Bank differed from that in Israel (10%); however, each location had similar co-trimoxazole (51%) resistance patterns. The spread of the co-trimoxazole–resistant clones is alarming because the World Health Organization has recommended use of this antimicrobial drug for treatment of nonsevere pneumoniae in children >2 months of age. No resistance was noted for cefotaxime, ofloxacin, and vancomycin (Table A1).

## Conclusions

Our study reports the distribution of *S. pneumoniae* serotypes in blood cultures of children residing in the West Bank. This study favors use of PCV13 because 80.8% vaccine coverage of the causative serotypes can be achieved compared with 50.8% with PCV7. In the West Bank, the increased serotype coverage of PCV13 in part results from inclusion of *S. pneumoniae* serotypes 1, 5, and 19A, which account for 23.4% of the serotypes not in PCV7. The introduction of PCV13 in the vaccination program will not only help reduce the incidence of IPD but will also help reduce infections caused by drug-resistant *S. pneumoniae* serotypes, such as serotype 14. Continuous monitoring of serotype distribution in this population will ensure that available vaccines can provide adequate coverage of circulating pneumococcal serotypes.

## Appendix

**Table A1:** Distribution of *Streptococcus pneumoniae* serotypes and antimicrobial susceptibility patterns, Palestinian Territories, West Bank*

Serotype	No. (%) isolates	Penicillin, %				Cefotaxime, %				Co-trimoxazole, %				Erythromycin, %				Ofloxacin, %				Vancomycin, %		
		S	I	R		S	I	R		S	I	R		S	I	R		S	I	R		S	I	R
**6A/B**	**17 (14.2)**	**100**	**0**	**0**		**100**	**0**	**0**		**52.9**	**11.8**	**35.3**		**47.1**	**5.9**	**47.1**		**100**	**0**	**0**		**100**	**0**	**0**
**14**	**16 (13.3)**	**87.5**	**13.5**	**0**		**100**	**0**	**0**		**18.8**	**6.2**	**75**		**25**	**0**	**75**		**87.5**	**12.5**	**0**		**100**	**0**	**0**
**1**	**14 (11.7)**	**100**	**0**	**0**		**100**	**0**	**0**		**7.14**	**35.7**	**57.1**		**92.9**	**7.1**	**0**		**100**	**0**	**0**		**100**	**0**	**0**
**9V**	**11 (9.2)**	**100**	**0**	**0**		**100**	**0**	**0**		**27.3**	**18.2**	**54.6**		**90.9**	**0**	**9.1**		**100**	**0**	**0**		**100**	**0**	**0**
**5**	**9 (7.5)**	**100**	**0**	**0**		**100**	**0**	**0**		**11.1**	**66.7**	**22.2**		**88.9**	**0**	**11.1**		**100**	**0**	**0**		**100**	**0**	**0**
**19F**	**8 (6.7)**	**100**	**0**	**0**		**100**	**0**	**0**		**37.5**	**12.5**	**50**		**62.5**	**0**	**37.5**		**100**	**0**	**0**		**100**	**0**	**0**
**4**	**6 (5.0)**	**100**	**0**	**0**		**100**	**0**	**0**		**83.3**	**0**	**16.7**		**66.7**	**16.7**	**16.7**		**100**	**0**	**0**		**100**	**0**	**0**
**19A**	**5 (4.2)**	**100**	**0**	**0**		**100**	**0**	**0**		**40**	**20**	**40**		**60**	**20**	**20**		**100**	**0**	**0**		**100**	**0**	**0**
Sg18	5 (4.2)	100	0	0		100	0	0		100	0	0		100	0	0		100	0	0		100	0	0
**3**	**4 (3.3)**	**100**	**0**	**0**		**100**	**0**	**0**		**50**	**0**	**50**		**25**	**25**	**50**		**100**	**0**	**0**		**100**	**0**	**0**
**7F**	**4 (3.3)**	**100**	**0**	**0**		**100**	**0**	**0**		**0**	**0**	**100**		**50**	**0**	**50**		**100**	**0**	**0**		**100**	**0**	**0**
8	3 (2.5)	100	0	0		100	0	0		0	66.7	33.3		100	0	0		100	0	0		100	0	0
**23F**	**3 (2.5)**	**100**	**0**	**0**		**100**	**0**	**0**		**0**	**0**	**100**		**100**	**0**	**0**		**100**	**0**	**0**		**100**	**0**	**0**
35B	3 (2.5)	100	0	0		100	0	0		100	0	0		100	0	0		100	0	0		100	0	0
12F	2 (1.7)	100	0	0		100	0	0		50	50	0		100	0	0		100	0	0		100	0	0
16F	2 (1.7)	100	0	0		100	0	0		100	0	0		100	0	0		100	0	0		100	0	0
33F	2 (1.7)	100	0	0		100	0	0		0	100	100		50	0	50		100	0	0		100	0	0
8	1 (0.8)	100	0	0		100	0	0		100	0	0		0	0	100		100	0	0		100	0	0
17F	1 (0.8)	100	0	0		100	0	0		100	0	0		100	0	0		100	0	0		100	0	0
38F	1 (0.8)	100	0	0		100	0	0		100	0	0		100	0	0		100	0	0		100	0	0
NT	3 (2.5)	100	0	0		100	0	0		100	0	0		100	0	0		100	0	0		100	0	0
Total	120	98.3	1.7	0		100	0	0		38.3	17.5	44.2		68.3	5	26.7		98.3	1.7	0		100	0	0
*S, susceptible; I, intermediate; R, resistant; NT, not serotyped. **Boldface** indicates pneumococcal conjugate valent 13 vaccine serotypes. Susceptibility testing and interpretation guidelines: penicillin MIC by Etest (S<2, I: 4, R>8 μg/mL); cefotaxime MIC by Etest (S<1, I 2, R>4 μg/mL); co-trimoxazole disk diffusion (S>19, I 16–18, R<15 mm); erythromycin disk diffusion (S>21, I 16–20, R<15 mm); ofloxacin disk diffusion (S>16, I 13–15, R<12 mm); vancomycin disk diffusion (>17 mm).																								
